# Local Brain Network Alterations and Olfactory Impairment in Alzheimer’s Disease: An fMRI and Graph-Based Study

**DOI:** 10.3390/brainsci13040631

**Published:** 2023-04-07

**Authors:** Bing Zhu, Qi Li, Yang Xi, Xiujun Li, Yu Yang, Chunjie Guo

**Affiliations:** 1 School of Computer Science and Technology, Changchun University of Science and Technology, Changchun 130022, China; 2 Jilin Provincial Key Laboratory for Numerical Simulation, Jilin Normal University, Siping 136000, China; 3 Zhongshan Institute of Changchun University of Science and Technology, Zhongshan 528437, China; 4 School of Computer Science, Northeast Electric Power University, Jilin 132012, China; 5 Department of Neurology and Neuroscience Center, The First Hospital of Jilin University, Changchun 130021, China; 6 Department of Radiology, The First Hospital of Jilin University, Changchun 130021, China

**Keywords:** Alzheimer’s disease, resting-state fMRI, olfaction, brain network, graph theory

## Abstract

Alzheimer’s disease (AD) is associated with the abnormal connection of functional networks. Olfactory impairment occurs in early AD; therefore, exploring alterations in olfactory-related regions is useful for early AD diagnosis. We combined the graph theory of local brain network topology with olfactory performance to analyze the differences in AD brain network characteristics. A total of 23 patients with AD and 18 normal controls were recruited for resting-state functional magnetic resonance imaging (fMRI), clinical neuropsychological examinations and the University of Pennsylvania Smell Identification Test (UPSIT). Between-group differences in the topological properties of the local network were compared. Pearson correlations were explored based on differential brain regions and olfactory performance. Statistical analysis revealed a correlation of the degree of cognitive impairment with olfactory recognition function. Local node topological properties were significantly altered in many local brain regions in the AD group. The nodal clustering coefficients of the bilateral temporal pole: middle temporal gyrus (TPOmid), degree centrality of the left insula (INS.L), degree centrality of the right middle temporal gyrus (MTG.R), and betweenness centrality of the left middle temporal gyrus (MTG.L) were related to olfactory performance. Alterations in local topological properties combined with the olfactory impairment can allow early identification of abnormal olfactory-related regions, facilitating early AD screening.

## 1. Introduction

Alzheimer’s disease (AD) is a multifactorial neurodegenerative disease that can cause the hidden progressive cognitive decline. Currently, more than 80% of patients with dementia have AD [1]. The clinical manifestations of AD include memory impairment, cognitive decline, aphasia, and executive dysfunction [2]. Approximately 40 million people worldwide have AD [3]. The pathological changes in AD are irreversible, and there is no effective treatment [4]. Therefore, diagnosing and managing patients with AD as early as possible is important to delay its progression [5].

Among various examinations, the most common diagnostic method for AD currently used is neuropsychological examination. This method is a simple, rapid, and standardized clinical detection testing tool. However, owing to the compensatory mechanism of brain injury, it is difficult to distinguish the early stage of AD from normal aging by neuropsychological examination, which greatly challenges an early clinical diagnosis.

The combination of graph theory and resting-state functional magnetic resonance imaging (fMRI) has become a powerful tool for studying brain separation and integration [6,7]. This method can quantitatively characterize the topological organization of brain networks [8,9]. For patients with neurological or psychiatric disorders, the resting-state fMRI can be easily performed without requiring stimulus presentation or patient response. Meanwhile, the brain can remain in a state of wakefulness and allow for relaxed movements during this period [10]. Through graph theory analysis of resting-state fMRI, lesions in certain localized brain regions may show alterations in the early functional network, causing a subtle impact on the life of patients with AD. The different property values of a local brain region reflect the connectivity of the different functions in this region, indicating the specific regions that have changed under the influence of disease. Node-based metrics can reflect changes in the response of tissues throughout the functional network and meticulously measure the nodal regions which are abnormally characterized during information transmission. Accordingly, the characteristics of the brain tissue have been revealed and the differences between healthy and diseased states compared [11,12], helping patients with targeted prognosis and treatment. This study aimed to provide a more objective clinical basis for early diagnosis of AD.

In terms of early biomarkers of AD, olfactory dysfunction is present in 85% of patients with early AD [13]. Therefore, it is an effective biomarker for early diagnosis. However, the olfaction-related regions which are abnormal during AD transformation remain unclear. In previous investigations, olfactory dysfunction and accelerated cognitive decline were related to the transition in AD status [14]. Recent studies have shown that odor-recognition tests can predict a decrease in cognitive ability [15,16]. This is a critical period in which the patient’s olfactory function has just shown abnormal signs without being judged as cognitive impairment. In this period, if the olfactory-related abnormal regions can be screened from many abnormal regions, it will provide early evidence for diagnosing and treating AD.

This study explored the early abnormal brain regions with significant differences in the brain network, combined with olfactory dysfunction in the early stages of AD, and examined the early abnormal brain regions related to olfaction. These observations aimed to provide objective information for the early detection of AD. To this end, the clinical neuropsychological examination and olfactory test results of patients with AD and healthy normal elderly individuals were compared. Based on the differences in the local network properties of the nodes, the key brain regions where abnormalities occurred were explored. Moreover, AD brain function abnormalities and their effects on daily physiological and cognitive activities were analyzed. We established a correlation between the olfactory test scores and the nodal properties with significant differences, and the diseased brain regions that may appear in the early stage related to the olfactory were screened.

## 2. Literature Review

Generally, the clinical diagnosis of AD is mainly based on the patient’s medical history of patients, clinical neuropsychological examination, and imaging. However, the compensatory mechanism of brain injury in patients poses great challenges to early clinical diagnosis. Searching for early biomarkers of AD and analyzing objective medical images have become the main research methods.

Many graph theory studies have focused on global network analysis during the resting state. This analysis method of the global network compared the small-world network properties, efficiency, normalized clustering coefficient, and other metrics to derive alterations in the integration of the entire brain network as the brain processes information [17]. Thus, from a global perspective, it can be concluded that the mechanisms of the functional organization are disrupted in patients with AD, and cognitive dysfunction may be caused by abnormal connectivity between different brain regions [18]. However, the specific regions that have changed in AD cannot be obtained from global-level analysis. Functional connectivity represents the temporal correlation between communication activities in different regions [19]. Some studies have used nodal degree and local efficiency as the main measures [20,21], focusing on functional interactions between various local levels of brain organization. Based on the calculation of the local network, Zhang et al. found that the betweenness centrality of the bilateral caudate nucleus and right superior temporal pole increased in patients with AD after treatment [22]. Therefore, the local network perspective in graph theory can be used to elucidate or gain insights into the characteristics of diseases.

Standardized behavioral examinations have shown that olfactory dysfunction may already be manifested in the prodromal stages of AD [23]. Roberts et al., through the comparison between baseline and follow-up diagnoses, found that cognitive impairment and odor identification testing were strongly associated, and the olfactory recognition impairment could be an early indicator of brain changes [24]. Devanand et al. suggested that patients with olfactory identification deficits, especially with those lacking awareness of olfactory deficits, are more likely to develop AD [25]. In addition, one hypothesis indicated that olfactory defects in AD are directly related to the underlying neuropathology of the early onset region [26]. Vasavada et al. considered that compared with visual, auditory, and somatosensory regions, lesions in local brain regions related to olfactory structures in pathology, such as the inferior medial temporal region, have existed in the earliest stages of the disease [27]. Accordingly, this study combined olfactory dysfunction with local brain network alterations to explore abnormal olfaction-related regions.

## 3. Materials and Methods

### 3.1. Participants

A total of 41 participants including 23 patients with AD and 18 normal controls (NC) were recruited and divided into two groups for this experiment. All participants provided voluntary informed consents according to the standards set by the Ethics Committee of the First Hospital of Jilin University. Furthermore, after obtaining a complete explanation of the experimental purpose and the basic procedure of the fMRI scan according to the protocol approved by the Institutional Research Review Committee, the participants provided written informed consent. In this study, a series of standardized clinical neuropsychological examinations were performed to evaluate the participants’ cognitive abilities. There were three participants in the AD group whose cognitive abilities were insufficient to complete the examinations and whose scores were not obtained. Therefore, they were not included in the statistical analysis. The Mini-Mental State Examination (MMSE) [28] has a full score of 30, which can be used for a simple cognitive assessment of patients with cognitive impairment. The Montreal Cognitive Assessment (MoCA) [29] is characterized by a simple process, short duration, and easy acceptance. The total score is 30 points, and a score of 26 or more indicates normal cognition. Impairment of memory and executive function is evaluated by Memory and Executive Screening (MES) with a total score of 100 [30]. Moreover, it should be noted that education level had little effect on the score. The Clinical Dementia Rating (CDR) [31] acts as an evaluation index of patient cognition; its score is represented by the range of 0 (no dementia), 0.5 (suspected), 1 (mild), 2 (moderate) and 3 (severe), respectively. The Boston Naming Test (BNT) [32] provides a comprehensive assessment of language ability, with scores greater than 22 considered normal. To explore the impact of differences in olfactory function among the two groups, we performed the University of Pennsylvania Smell Identification Test (UPSIT) [33] assessment on the participants. On a scale of 40, 35–40 points indicate normal smell, 31–34 points indicate mild olfactory dysfunction, 26–30 points indicate moderate olfactory dysfunction, and 19–25 points indicate severe olfactory dysfunction. Demographic and neuropsychological examinations were conducted individually for each participant by professional evaluators of neuropsychological research.

### 3.2. fMRI Data Acquisition

The resting-state fMRI data were collected using a Philips Ingenia 3.0T scanner at the First Hospital of Jilin University. During this process, each participant was required to avoid head movement and close their eyes without thinking or falling asleep. It took 8 min for each scan of the 240 functional volumes to acquire the fMRI data. The specific parameters were as follows: repetition time (TR) = 2000 ms; echo time (TE) = 30 ms; flip angle (FA) = 90°; field-of-view (FOV), 224 × 224 × 138 mm^3^; matrix size, 64 × 63 mm^2^; slice thickness = 3.5 mm, and 33 slices. All original image files were available for subsequent analysis.

### 3.3. Data Preprocessing

fMRI data were preprocessed using the Statistical Parametric Mapping (SPM12, http://www.fil.ion.ucl.ac.uk/spm/, accessed on 8 June 2020) and the Data Processing Assistant for Resting-State fMRI (DPARSF, http://rfmri.org/DPARSF, accessed on 20 June 2020). The first 10 time points were discarded to eliminate the initial transit signal fluctuations and reduce participants’ adaptation to the circumstances. The following functional images were processed through a standardized data preprocessing process, including a slice-timing correction to correct the difference in acquisition time between layers of a volume and the realignment of head movement with the first volume. Due to the inclusion of the patients, the participants’ head movements were controlled at 3.0 mm in any dimension and 3.0° in any direction. The remaining images were spatially normalized to the Montreal Neurological Institute (MNI) space with an Echo Planar Imaging (EPI) template and resampled to a voxel size of 3 × 3 × 3 mm^3^. The images were smoothed with a Gaussian kernel of 8 mm full width at half maximum (FWHM) to decrease spatial noise. The linear trends removed movement-related noise after realignment or instrumental instability. Finally, temporal bandpass filtering (0.01 Hz ≤ f ≤ 0.1 Hz) was performed to reduce the effects of low-frequency drift and high-frequency noise.

### 3.4. Construction of Functional Brain Network

Functional brain networks were constructed based on the regions of interest (ROIs). An anatomical automatic labeling (AAL, http://www.cyceron.fr/freeware/, accessed on 11 January 2021) brain template was used to parcellate the brain into 90 regions of the cerebrum and 26 regions of the cerebellum (Table 1 shows part of the brain region abbreviations). Here, we mainly describe the regions of the cerebrum. These regions were formed by applying multiple linear regression analysis and functional connection mapping to processed resting-state fMRI data. Subsequently, a mean time series was extracted from 90 cerebral regions, and the Pearson correlation coefficient was calculated to estimate the functional correlation among the time series [34]. Each participant generated a 90 × 90 correlation matrix. The correlation matrix was thresholded and was set at 0.10 to 0.50 with a partition interval of 0.01 to obtain a binarized matrix. A graphical model of each participant’s brain functional network was constructed using a binary matrix. We explored the functional relationships between the brain regions by analyzing these binary matrices.

### 3.5. Network Analysis

Topological properties of the corresponding brain networks were determined using the graph theoretical network analysis toolbox (GRETNA, http://www.nitrc.org/projects/gretna, accessed on 16 February 2022). Through graph theory, network architecture was used to analyze the nodal metrics of the resting-state fMRI. Nodal local efficiency, nodal efficiency, nodal clustering coefficient, degree centrality, and betweenness centrality were calculated to evaluate the local characteristics of each cortical region in the functional networks of the two groups. Nodal local efficiency refers to information dissemination among a node’s direct neighbors, reflecting the network’s fault tolerance. The nodal efficiency represents the average difficulty of a node from other nodes in the network. The higher the efficiency of a node, the easier it is to transmit information to the other nodes. The clustering coefficient of a node is used to measure the degree of network clustering and is related to neighboring communication. This measures the possibility that the neighbors of a node are neighbors. Degree centrality describes the centrality of a node in the network; the node with the largest degree is considered the core node of the network. Degree centrality reflects the importance of a node within a network. Similar to nodal degree centrality, another parameter used to describe the importance of a node in a network is betweenness centrality. This is a related and usually more sensitive metric, which is defined as the fraction of all the shortest paths in the network that pass through a given node. It is a demonstration of their ability to connect [35,36].

### 3.6. Statistical Analysis

Statistical analysis was performed using SPSS 25 software (IBM Statistical Package for the Social Sciences, Inc., Chicago, IL, USA). The calculation of network properties did not depend on a specific threshold, and the network sparsity thresholds were selected from 0.10 to 0.50. Statistical analysis was performed by calculating the average values of the area under the curve. Topological measurements of the two groups were statistically compared using a two-tailed independent sample *t*-test. A two-tailed *χ*^2^ test was used for gender comparison. Pearson correlation analysis was used to determine the correlation coefficients between the two sets of scales or ordinal variables. However, when the data were identified as being non-normally distributed, Spearman correlation analysis was used. The correlation calculations included the correlations between the neuropsychological measures and UPSIT scores as well as correlations between topological measurements with significant differences and UPSIT scores. A value of *p* < 0.05 was considered a statistically significant difference.

## 4. Results

### 4.1. Demographic and Neuropsychological Measures

Table 2 provides the demographic and neuropsychological summary of the AD and NC groups, and no significant differences were found in age, gender or education level between the two groups. The neuropsychological examinations (MMSE, MoCA, MES, CDR, and BNT) showed significant differences between the two groups (independent sample *t*-test, all *p* < 0.001). The average scores of the NC group in the MMSE, MoCA, MES, and BNT groups were higher than those of the AD groups. Based on the CDR results, the average score of the AD group was between mild and moderate cognitive impairment, while the NC group had normal cognition. Moreover, the UPSIT scores of the groups of participants were significantly different, with the AD group having lower scores than the NC group.

### 4.2. Relationships between UPSIT Scores and Neuropsychological Measures

**Figure 1:**
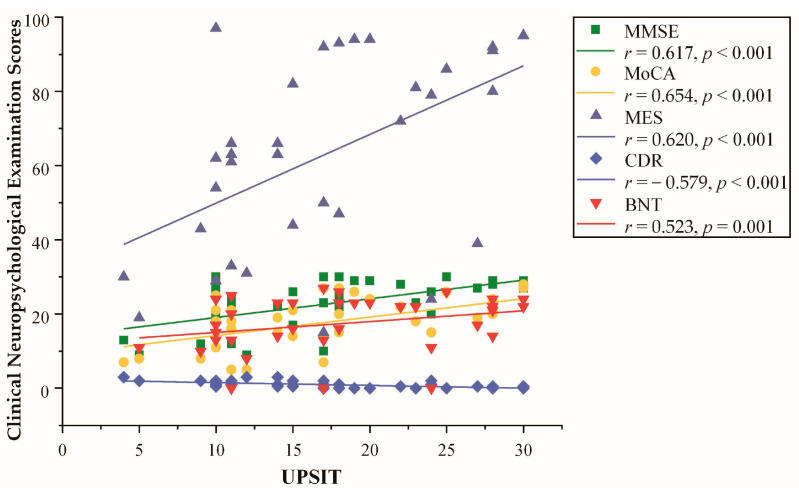
Pearson correlation between the University of Pennsylvania Smell Identification Test (UPSIT) scores and clinical neuropsychological examination scores in two groups; *p* < 0.05 was the statistical test analysis result of Pearson correlation.

Pearson correlations between the UPSIT scores and neuropsychological measures are shown in Figure 1. UPSIT scores were correlated with all five neuropsychological measures, which were positively correlated with MMSE (*r* = 0.617, *p* < 0.001), MoCA (*r* = 0.654, *p* < 0.001), MES (*r* = 0.620, *p* < 0.001), and BNT (*r* = 0.523, *p* = 0.001) scores, and they were negatively correlated with CDR (*r* = −0.579, *p* < 0.001) scores.

### 4.3. Nodal Properties

**Figure 2:**
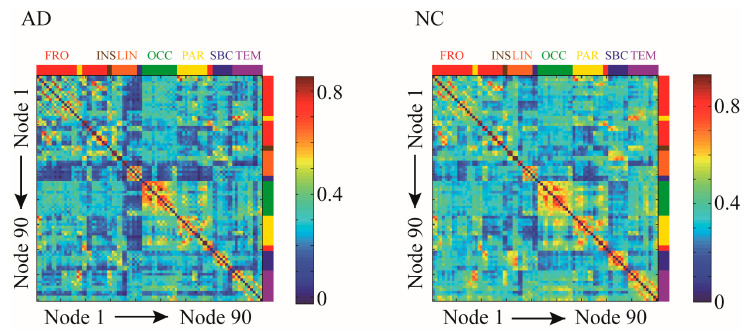
Correlation matrices of the functional brain networks in two groups. The correlation matrices of the brain function networks of group Alzheimer disease (AD) and normal controls (NC) were calculated based on 90 regions in the AAL90 template. The brain regions of the AAL90 template are assigned, where red represents the frontal lobe, yellow represents the parietal lobe, green represents the occipital lobe, purple represents the temporal lobe, brown represents the insula, orange represents the limbic system and blue represents the subcortex.

**Figure 3:**
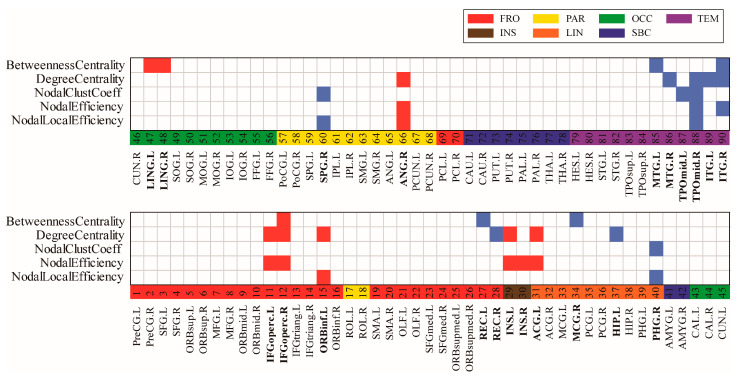
The regions with significant differences in nodal properties between groups; *p* < 0.05. The significant increase and decrease in node network indicators are marked in red and blue, respectively, indicating that the two groups of nodes in AAL90 were analyzed using a two-tailed independent sample *t*-test in nodal local efficiency, nodal efficiency, nodal clustering coefficient, degree centrality and betweenness centrality. The brain lobes were marked with different colors: red for the frontal lobe, yellow for the parietal lobe, green for the occipital lobe, purple for the temporal lobe, brown for the insula, orange for the limbic system and blue for the subcortex.

**Figure 4:**
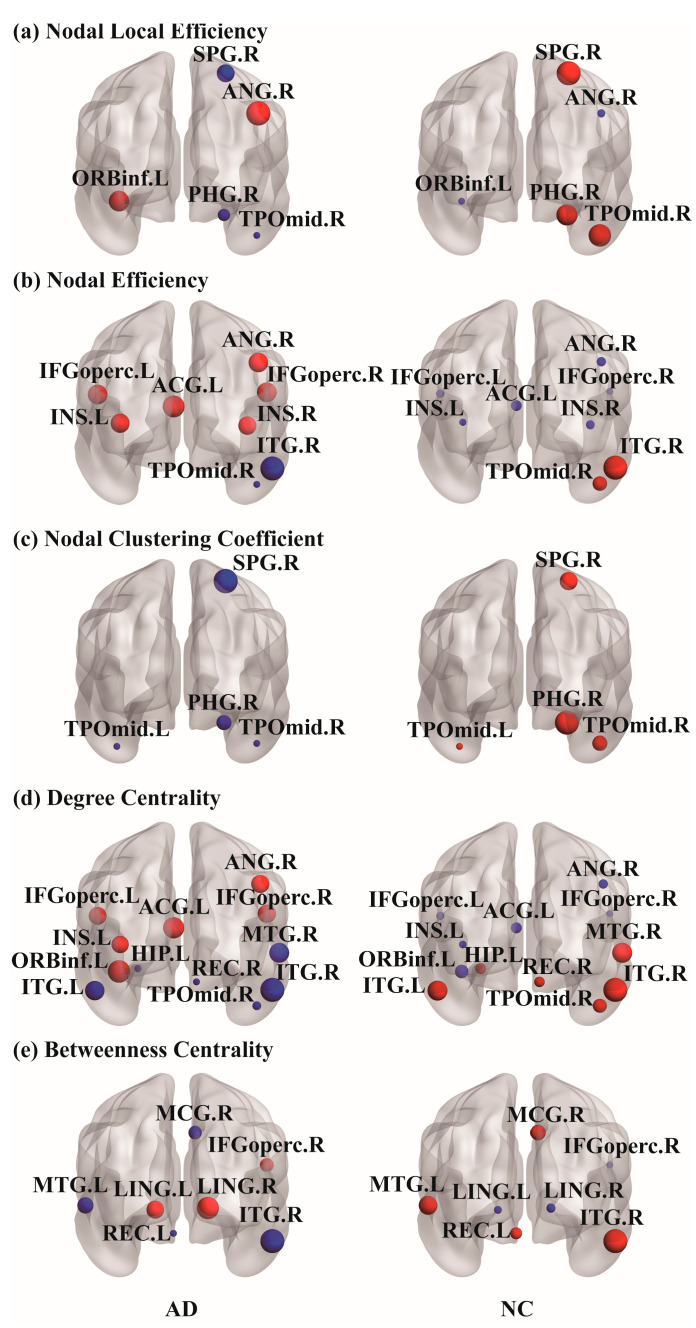
Plots of regions on the cortical surface area. In AAL90, (**a**) nodal local efficiency, (**b**) nodal efficiency, (**c**) nodal clustering coefficient, (**d**) degree centrality and (**e**) betweenness centrality nodal properties have significant differences between the two groups; *p* < 0.05 was the statistics of two-tailed independent sample *t*-test analysis results. When comparing each property, red and blue represent the increase or decrease in node property values. The size of the sphere represents the property value. Using BrainNet Viewer software, nodes are mapped to the cortical surface.

The correlation matrices and functional connectivity patterns of the two groups are shown in Figure 2. The nodal properties of the functional brain networks were calculated based on these correlation matrices. To investigate the alterations in whole-brain node metrics between the two groups, a two-tailed independent sample *t*-test was used. Figure 3 presents the results of the five nodal properties, of which 21 nodes showed significant differences. Five nodal local efficiency calculation results were obtained: ORBinf.L, PHG.R, SPG.R, ANG.R, and TPOmid.R. In PHG.R, SPG.R, and TPOmid.R, the nodal local efficiency values in the AD group were lower than those in the NC group. Eight significant differences were found in the nodal efficiency calculation results: bilateral IFGoperc, bilateral INS, ACG.L, ANG.R, TPOmid.R, and ITG.R. Except for the TPOmid.R and ITG.R regions in the temporal lobe, nodal efficiency in the AD group was higher than those in the NC group. There were four significant differences in the calculation results of the nodal clustering coefficient: PHG.R, SPG.R, and bilateral TPOmid. The values in the AD group were all lower than those in the NC group. Through calculations, it was found that the 12 nodal regions had significant differences in degree centrality. These were bilateral IFGoperc, ORBinf.L, REC.R, INS.L, ACG.L, HIP.L, ANG.R, MTG.R, TPOmid.R, and bilateral ITG. In the statistical results, the AD group’s REC.R, HIP.L, MTG.R, TPOmid.R, and bilateral ITG degree values were smaller than the NC group. On the contrary, the other six regions with significant differences were all larger than the NC group. Seven were counted in betweenness centrality, IFGoperc.R, REC.L, MCG.R, bilateral LING, MTG.L, and ITG.R. Among them, four values, REC.L, MCG.R, MTG.L, and ITG.R were lower in the AD group than in the NC group, and the other three values were greater in the AD group than in the NC group. It should be noted that the TPOmid.R region had four significant differences in the statistical results for the five properties. Moreover, the significantly different nodes appearing in the temporal lobe region and the property values of the AD group were all smaller than those of the NC group. The locations of these nodal regions in the brain are shown in Figure 4.

### 4.4. Relationships between the UPSIT Scores and Nodal Properties

**Figure 5:**
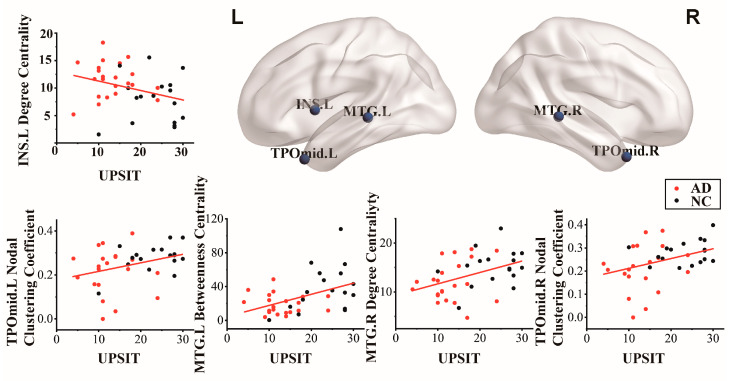
Plots of regions on the cortical surface with significant nodal properties regions in the two groups and University of Pennsylvania Smell Identification Test (UPSIT) scores in the Pearson correlation brain region; *p* < 0.05 was the statistical test analysis result of Pearson correlation.

Pearson correlation analysis was used to calculate the relationship between the UPSIT scores and the five nodal properties, and significant differences were found between the two groups. As shown in Table 3, the correlation between the UPSIT scores and nodal properties was significantly different between the AD and NC groups. Among these, five nodal properties were correlated with UPSIT scores. Node TPOmid.L (*r* = 0.320, *p* = 0.047) and TPOmid.R (*r* = 0.383, *p* = 0.016) from the nodal clustering coefficient were positively correlated with UPSIT scores. In evaluating degree centrality and UPSIT scores, there was a negative correlation in the INS.L (*r* = −0.317, *p* = 0.049) and a positive correlation with the MTG.R (*r* = 0.417, *p* = 0.008). In addition, the MTG.L (*r* = 0.446, *p* = 0.004) of betweenness centrality was related to UPSIT scores. Figure 5 shows the cortical surface and related scatterers, corresponding to Pearson-related nodes in the AD and NC groups.

## 5. Discussion

Significant differences were found between the AD and NC groups in the scores of neuropsychological statistics and UPSIT olfactory detection scores, and the two types of scores showed a correlation trend. Combined with graph theory analysis of resting-state fMRI, specific brain functional network differences between brain regions were calculated, which were characterized by changes in different properties. Moreover, the relationship between local cortical regions that showed differences in olfactory performance was also explored.

### 5.1. Decrease in Cognitive Level and Olfactory Function

The AD and NC groups showed significant differences in neuropsychological examinations and UPSIT scores, confirming the decline in the cognitive and olfactory identification in AD. Although several neuropsychological evaluation scores were different, the results showed that the AD group’s cognitive level decreased significantly in learning, memory, executive function, and language expression. As depicted in Figure 1, the relevance of the associated with the results of UPSIT score and neuropsychological examinations showed that the olfactory recognition function gradually declined with the aggravation of cognitive impairment. This indicates that odor recognition examinations have potential use in screening for AD. They can also be used for the early detection of people at cognitive risk. In summary, the olfactory recognition function can be used as a sensitive indicator for the assessing of AD [24,37,38].

### 5.2. Alteration in Nodal Network Topology

The nodal network topology measurement method can be used to evaluate the importance of a single node region for different nodal properties. This study used five nodal properties to compare the two groups in a multifaceted manner for local region transitions. Compared with a single property, incorporating different connectivity metrics into the network analysis of the characteristics can help complement information and capture regional characteristics more comprehensively. Significant alterations in some functional regions existed for each of the five nodal properties. The alterations were widely distributed in most cortical regions and spread throughout the cerebral cortex. The increases and decreases in the two groups of values in the same region were different. As shown in Figure 4, the clustering coefficient values of nodes affected by the AD group tended to decrease compared to those of the normal participants. As an index to measure the degree of network clustering, a decrease in the clustering coefficient also indicates that the connection between the node and neighboring nodes is decreasing, and the information transmission capacity is also weakened. These regions can continue to integrating local functions to maintain the balance of brain operations. As shown in Figure 3, the obtained results were similar to those of previous studies, and some regions in the frontal and temporal lobes showed significant characteristic increases and decreases [39]. These regions with significant differences will become our focus.

Recent studies have reported that increased functional connectivity in the frontal lobe is associated with the maintenance of episodic memory [40,41,42]. According to the calculated results in Figure 3, there were significant differences between the two groups in the bilateral IFGoperc and ORBinf.L of the inferior frontal gyrus. The values in the AD group were greater than those in the NC group. It is widely accepted that IFGoperc is involved in the maintenance of memory [43,44] and odor recognition [23]. Based on the above results, the bilateral IFGoperc showed significant alterations due to the influence of AD. The connections with other regions increased, and the information transmission capacity improved. Meanwhile, the degree of collectivization decreased, and the status and role of the network increased. Moreover, it is well known that the inferior frontal gyrus could participate in the maintenance of memory, involving the integration of information accompanied by the encoding and unlocking of episodic memory [41]. The inferior frontal gyrus plays a key role in interference and helps to maintain cognitive control processes. In addition, some studies suggest that REC is associated with alexithymia, cognitive empathy, and successful memory performance [45]. In conclusion, it was confirmed that some frontal gyrus regions could be affected by AD, playing an important role in studying the effect of AD on memory.

The AD and NC groups showed significant differences in the temporal lobe’s bilateral MTG, bilateral TPOmid, and bilateral ITG. These regions in the AD group showed a downward trend compared to those in the NC group. These regions involve high-level cognitive functions such as episodic memory, attention, motivation, self-awareness, and audiovisual integration [46]. The effect of AD on the temporal lobe has been proven in many documents [47,48]. For example, Dai et al. found that the AD group had a reduced degree centrality of nodes in the right superior temporal pole, TPOmid.R, and ITG.R [49]. Cognition refers to how people acquire, process, and apply information through perception. Alterations in temporal lobe regions are associated with the appearance of objective cognitive impairment [47]. This was also consistent with the results of the clinical neuropsychological examinations. Moreover, the temporal pole is known to be involved in recognizing olfactory odors and regulating different cognitive functions, such as attention, recognition, emotion, and memory [20]. Notably, in Figure 3, we found that the two groups were present in the regional assessment of the four properties of TPOmid.R, indicating that TPOmid.R plays a considerable role in the brain topology network and is the potential center of the neural substrates related to cognitive reserve. In general, compared to other lobes in AD, the topological network properties of the partial regions in the temporal lobe decreased significantly. This could provide valuable information for the development and early diagnosis of AD.

AD is characterized by memory and cognitive impairment, which is related to the observed abnormalities in HIP, PHG, and other related regions. In this study, our results showed that some limbic system regions altered network topology, including HIP.L, PHG.R, ACG.L, and MCG.R. In a previous histopathological study, the limbic system degenerated in the early stages of AD [50]. Moreover, Pili et al. pointed out that HIP is one of the earliest brain regions to be altered in AD, and that the extent of abnormalities may reflect the disease severity [51]. It is responsible for encoding long-term memories and assisting spatial navigation in AD. HIP.L showed a decrease in degree centrality compared to NC. The degree of coupling in the HIP.L of AD was disrupted. The function of the PHG mainly involves memory creation and the recall of visual scenes. PHG.R of AD showed a decrease in the nodal local efficiency and clustering coefficient. The communication and information transmission efficiency of the PHG.R was decreased. The cingulate region is the first to be affected by AD [52]. ACG in the limbic system has subregional differences in many previous neuropsychiatric and neurodegenerative diseases brain network studies [53,54]. The increase in the regional activities of ACG.L could be interpreted as a mechanism compensating for olfactory memory tasks. MCG.R is the key region for proactive rather than reactive action control. The betweenness centrality of this region decreased, indicating that the control ability also decreased. In addition, the INS is thought to be involved in emotional processing and arousal, including awareness of one’s physical state, decision making, and other executive processes [55]. INS is also believed to direct the regulation of cerebral circulation, thereby helping maintain memory [56,57]. The increase in nodal efficiency and the topology with surrounding nodes all prove that the INS had disequilibrium in the control of sensation, memory, and visceral control. The bilateral LING in the occipital lobe is closely related to visual recognition and plays an important role in episodic memory [58]. In the parietal lobe, SPG involves discerning aspects of sensation, such as shape, roughness, size, texture and memory of the position of an object in space [59]. ANG is related to language processing, mathematics, and other cognitive skills. In summary, the results obtained are consistent with the clinical manifestations of AD. The abnormalities in these regions may indicate disease progression and confirm the demographic and neuropsychological results.

### 5.3. Interaction between Olfactory Performance and Nodal Alteration Region

Olfactory ability is closely associated with brain function and health. However, alterations in olfactory abilities are often overlooked. Researchers believed that the risk of cognitive decline above the olfactory performance threshold is reduced in elderly people [60]. Studies of AD olfactory disorders found that the impairment of olfactory-related regions [61,62] could not completely cause the anosmia in the olfactory function of patients with AD. There remains residual capacity for additional recruitment. Impairment of odor identification is an early sign of the brain losing its ability to repair itself. Therefore, after obtaining the abnormal brain regions, the UPSIT scores for selecting or stratifying were calculated using Pearson correlation to screening the main regions with olfactory impairment [63].

The results in Table 3 displayed that the local nodal properties with significant differences in correlation with UPSIT scores in the two groups were mainly found in the INS.L and local regions of the temporal lobe. This proved that the function of some local regions in the AD group had changed compared with the normal elderly group, leading to a decline in olfactory function. The degree centrality of INS.L was negatively correlated with UPSIT scores, and the degree centrality values of INS.L in the AD group were higher than those in the NC group. INS is involved in the perceptual processing of odor characteristics. Previous order fMRI studies have shown a significant increase in INS activation during the response to pleasant and unpleasant odors [64,65]. This suggests that the INS responds to the hedonics of odors and is involved in processing the emotional aspects of odors. This reflects the role of the INS.L region in olfaction. Although the olfactory function of patients with AD showed a downward trend in the UPSIT, it prompted the improvement of INS.L in olfactory recognition analysis. The alteration of INS.L nodal properties may have been a key local region in the early stages of AD.

Middle temporal lobe structures are well known to be significantly atrophic in AD [66,67]. Moreover, bilateral MTG is associated with odor memory, odor recognition, and odor processing [68]. Accordingly, the correlation between the decreased property values of the bilateral MTG region and UPSIT scores in the AD group confirmed a decrease in olfactory function. In addition, in neuroimaging literature, the temporal pole can connect the emotional processing of olfactory stimuli with mentalizing or theory of mind and combine emotional responses with highly processed sensory stimuli. The temporal pole collects information obtained by the sense of smell, perceives and remembers the acquired information, and makes visceral emotional responses [49,69]. The memory function of this region allows the storage of perception–emotion connections, forming the basis of personal semantic memory. As shown in Table 3, the bilateral TPOmid in the two groups was associated with olfactory processing and feedback. However, the decreasing trend of UPSIT scores and nodal clustering coefficient values of bilateral TPOmid in the AD group confirmed that patients with AD had different performances in odor discrimination. Therefore, the study of olfactory-related alterations in local brain regions may be useful for improving the early discovery of AD and may further promote the early treatment interventions for AD.

### 5.4. Limitations

Although the current research results have confirmed that AD affects the alterations in regional brain function and olfactory-related brain network dysfunction, this study has some limitations. First, in terms of data collection, the amount of data collected from the participants was not large. Here, we only compared and analyzed participants in the AD and NC groups. In the future, we will continue to collect data and try to recruit participants with intermediate transition states, such as subjective cognitive decline and mild cognitive impairment, to explore the gradual progression of AD. In addition, we plan to increase the task fMRI experimental design. More in-depth research on the olfactory system should be carried out. The combination of olfactory detection and fMRI can provide more objective evaluation information for exploring early biomarkers of AD from the behavioral and imaging aspects. Furthermore, this approach could also be considered for studying neurodegenerative diseases such as dementias and tauopathies.

## 6. Conclusions

We applied graph theory to analyze the differences in the resting-state fMRI brain network between patients with AD and healthy elderly individuals as well as the difference in the effect of alterations in olfactory function on local brain regions. Five nodal properties were used to evaluate abnormalities in local regions. The calculated local regions with significant differences correlated with the UPSIT scores; that is, the local regions related to olfaction that may appear in the early stage of AD were obtained. Our study suggests that as the nodal properties of many local regions are altered, there is a gradual decrease in olfactory discrimination and an increase in cognitive impairment. In summary, olfactory dysfunction is an early symptom of AD. Combining a simple, quick, and cost-effective olfactory test with localized abnormal functional brain regions will provide new directions for early diagnosis and more time for patient treatment.

## Figures and Tables

**Table 1:** Abbreviations for brain regions.

Abbreviation	Region Name
ACG	Anterior cingulate and paracingulate gyri
ANG	Angular gyrus
HIP	Hippocampus
IFGoperc	Inferior frontal gyrus, opercular part
INS	Insula
ITG	Inferior temporal gyrus
LING	Lingual gyrus
MCG	Median cingulate and paracingulate gyri
MTG	Middle temporal gyrus
ORBinf	Inferior frontal gyrus, orbital part
PHG	Parahippocampal gyrus
REC	Gyrus rectus
SPG	Superior parietal gyrus
TPOmid	Temporal pole: middle temporal gyrus

**Table 2:** Demographic and neuropsychological data of the study participants.

	AD (*n* = 23)	NC (*n* = 18)	*t*/*χ*^2^	*p*
Age	66.69 ± 7.03	63.44 ± 8.16	1.368 *	0.179
Gender (M/F)	8/15	4/14	0.770 ^#^	0.380
Education	10.95 ± 4.18	10.94 ± 3.78	0.010 *	0.992
MMSE	18.76 ± 5.82	27.82 ± 2.18	−6.583 *	*p* < 0.001
MoCA	13.47 ± 5.21	23.29 ± 3.23	−7.105 *	*p* < 0.001
MES	45.47 ± 17.94	85.70 ± 13.89	−7.579 *	*p* < 0.001
CDR	1.59 ± 0.78	0.20 ± 0.25	7.800 *	*p* < 0.001
BNT	12.40 ± 7.75	22.58 ± 3.16	−5.373 *	*p* < 0.001
UPSIT	13.13 ± 5.09	23.23 ± 5.87	−5.742 *	*p* < 0.001

AD, Alzheimer’s disease. NC, normal controls. MMSE, Mini-Mental State Examination; MoCA, Montreal Cognitive Assessment; MES, Memory and Executive Screening; CDR, Clinical Dementia Rating; BNT, Boston Naming Test; UPSIT, University of Pennsylvania Smell Identification Test. *, two-tailed independent sample *t*-test; *p*, *p*-value; ^#^, *χ*^2^ test.

**Table 3:** UPSIT scores effects on nodal regions.

Properties	Regions	*r*	*p*
Nodal Clustering Coefficient	TPOmid.L	0.320	0.047
Nodal Clustering Coefficient	TPOmid.R	0.383	0.016
Degree Centrality	INS.L	−0.317	0.049
Degree Centrality	MTG.R	0.417	0.008
Betweenness Centrality	MTG.L	0.446	0.004

The *p*-value obtained through Pearson correlation.

## Data Availability

Not applicable.
